# Characterizing barriers to antibiotic stewardship for skin and soft-tissue infections in the emergency department using a systems engineering framework

**DOI:** 10.1017/ash.2022.316

**Published:** 2022-11-07

**Authors:** Michael S. Pulia, Rebecca J. Schwei, Steven P. Hesse, Nicole E. Werner

**Affiliations:** 1 BerbeeWalsh Department of Emergency Medicine, University of Wisconsin—Madison School of Medicine and Public Health, Madison, Wisconsin; 2 Department of Industrial and Systems Engineering, University of Wisconsin—Madison, Madison, Wisconsin; 3 University of Wisconsin—Madison School of Medicine and Public Health, Madison, Wisconsin

## Abstract

**Objective:**

Skin and soft-tissue infections (SSTIs) account for 3% of all emergency department (ED) encounters and are frequently associated with inappropriate antibiotic prescribing. We characterized barriers and facilitators to optimal antibiotic use for SSTIs in the ED using a systems engineering framework and matched them with targeted stewardship interventions.

**Design and participants:**

We conducted semistructured interviews with a purposefully selected sample of emergency physicians.

**Methods:**

An interview guide was developed using the Systems Engineering Initiative for Patient Safety (SEIPS) framework. Interviews were recorded, transcribed, and analyzed iteratively until conceptual saturation was achieved. Themes were identified using deductive directed content analysis guided by the SEIPS model.

**Results:**

We conducted 20 interviews with physicians of varying experience and from different practice settings. Identified barriers to optimal antibiotic prescribing for SSTIs included poor access to follow-up (organization), need for definitive diagnostic tools (tools and technology) and fear over adverse outcomes related to missed infections (person). Identified potential interventions included programs to enhance follow-up care; diagnostic aides (eg, rapid MRSA assays for purulent infections and surface thermal imaging for cellulitis); and shared decision-making tools.

**Conclusions:**

Using a systems engineering informed qualitative approach, we successfully characterized barriers and developed targeted antibiotic stewardship interventions for SSTIs managed in the ED work system. The interventions span multiple components of the ED work system and should inform future efforts to improve antibiotic stewardship for SSTIs in this challenging care setting.

Antibiotics are unique therapeutic agents often referred to as “societal” medications due to their ability to simultaneously affect the patient being treated and the community at large.^1,2^ Inappropriate use of antibiotics in healthcare settings is most often characterized as resulting from a failure to adhere to best-practice guidelines and/or diagnostic error. This gap in care quality has been identified as a primary, modifiable contributor to the global increase in antibiotic-resistant bacterial infections.^3^ Thus, there have been multiple “calls to action” related to antibiotic stewardship, including those targeting the emergency department (ED).^4^

Skin and soft-tissue infections (SSTIs) account for ∼3% of all ED encounters (>3 million annual visits), and inappropriate antibiotic prescribing for this condition occurs frequently in this setting.^5–8^ There is a clear need to identify interventions that can optimize antibiotic use in the management of SSTIs in the ED. To successfully improve prescribing, interventions must be informed by key drivers of behavior, which can vary by provider type and setting.^9^ Although much is known about drivers of guideline-discordant antibiotic use for other conditions (eg, respiratory tract infections) and settings, the literature for SSTIs and the ED setting is comparatively limited.^10,11^

The International Federation for Emergency Medicine published a report characterizing the ED as a unique clinical environment regarding quality and safety interventions.^12^ The report emphasizes the need for human factors and systems engineering informed approaches to successfully overcome these barriers. Therefore, we sought to characterize barriers and facilitators to optimal antibiotic use in the management of SSTIs in the ED using a systems engineering framework and to match them with targeted stewardship interventions.

## Methods

### Sampling

We conducted semistructured interviews at a national emergency medicine (EM) conference. To achieve conceptual saturation, we conducted additional interviews with EM physicians working at university and community EDs in the Midwest.^13^ To be eligible, physicians needed to be actively practicing clinical EM in the United States and have completed or be in the final year of an EM residency program. All participants received $100 in financial incentive following the interview.

We recruited participants through conference e-mails and brochures included with conference materials. We also recruited participants from EDs in Wisconsin by direct e-mails. We selected potential participants by purposeful criterion sampling to ensure that perspectives from a range of settings (ie, urban, suburban, rural), geographic locations, years of experience, sex, and size of the ED were represented.^14^ Interviews and analyses were conducted over a 2-year period spanning 2017–2019. Our institutional review board approved all study activities.

### Design and procedure

Using semistructured interviews, we explored broad themes around the diagnostic and antibiotic decision-making process for SSTIs that would be applicable across practice settings. Interview questions were primarily open ended so the participant could respond with what came to mind first. Probing follow-up questions were based on elements of the Systems Engineering Initiative for Patient Safety (SEIPS) framework and were utilized to identify themes within each element of the framework (see Supplement 1: Interview Guide). SEIPS was developed to comprehensively assess elements of healthcare work systems that affect care processes and outcomes. SEIPS has been successfully applied to characterize various quality of care and patient safety challenges (eg, antibiotic stewardship and diagnostic errors).^15–17^ The element at the center of the model is the person (provider or patient) with surrounding elements (ie, physical environment, tasks, organization, and tools and technology) that operate within an external environment. The elements of the work system interact when preforming healthcare processes, which produce outcomes that feed back into the work system.

A nonclinical, study-team member with 5 years of experience in qualitative methods (R.J.S.) conducted one-on-one interviews in a private room. The principle investigator, a practicing EM physician with advanced training in systems engineering and qualitative methods (M.S.P.), attended 2 of the initial interviews to observe, ask additional clarifying questions, and facilitate minor modifications of the interview guide. We pilot tested the semistructured interview guide with 2 EM physicians at our institution. As interviews progressed, we refined questions and incorporated more pointed follow-up questions to encourage physicians to elaborate on the emerging themes.

We audio recorded all interviews, and a private company professionally transcribed audio files verbatim, which the study team reviewed for accuracy. Prior to starting the interview, we asked physicians demographic (eg, sex and years of experience) and practice-setting questions (eg, type of ED, teaching versus nonteaching, the annual ED volume per year, and the geographic region of the country where they worked). We proceeded with sampling, data collection and data analysis concurrently. We stopped collecting data when sufficient heterogeneity in participant answers was achieved as indicated by the responses becoming redundant and targeted probes failing to uncover new themes (ie, conceptual saturation).^13^

### Content analysis

We used deductive directed content analysis guided by the SEIPS model.^18^ Researcher R.J.S. wrote an initial memo after each interview to capture emerging concepts and general observations; we used these memos as we generated the code book.^19^ The study team developed a preliminary code book based on the domains of the interview questions and the elements in the SEIPS model.^20^ Two study team members (M.S.P. and R.J.S.) used the preliminary code book and coded 6 interviews independently. Next, the coders met to review codes, add new codes, and refine code definitions. The study team continued to use memos during the coding process to track how code definitions evolved and to track divergent cases. We conducted this process for 6 interviews. For the remaining interviews, R.J.S. completed primary coding and M.S.P. conducted a secondary review, adding codes as needed. Any discrepancies in coding were resolved by discussion and consensus.^21^ The finalized code book is included in Supplement 2. We used Dedoose, qualitative data software, to facilitate the coding process.^22^

### Intervention development

Once coding was complete, we generated a list of codes representing potentially modifiable barriers and facilitators or strategies. Following established intervention development methods, each identified modifiable barrier and/or facilitator or strategy was matched with a proposed intervention.^23,24^ The interventions were then presented to a diverse group of 12 stakeholders from the author’s affiliated healthcare system in a series of either small group (n = 3) or individual meetings (n = 5) to elicit feedback. Stakeholders were identified and verbally invited to participate. The group was selected based on a goal of having diverse and multidisciplinary perspectives considered. As such, the group included 3 emergency physicians, 1 emergency medicine resident, 1 mid-level emergency provider, 2 ED nurses, 1 member of the ED clinical operations team, 2 infectious diseases physicians, and 2 infectious diseases pharmacists on the hospital antimicrobial stewardship committee. The meetings ranged from 20 to 40 minutes in length, and suggested revisions on the structure of the proposed interventions were captured by detailed notetaking. Revisions of the interventions and associated descriptions continued until there was group consensus that no further edits were necessary.

## Results

In total, 39 physicians expressed interest in participating, and we conducted 20 interviews. No one refused to participate. The average interview lasted 48 minutes. The demographic and practice-setting characteristics are summarized in Table 1. Purposeful sampling yielded representation from a range of settings (urban, suburban, rural), geographic locations, years of experience, sex, and size of ED. The results that follow are organized according to the primary work system code of the SEIPS model with the last section of the results describing cross-cutting themes and targeted interventions proposed.

**Table 1.** Description of Physician and Practice Setting Characteristics (n=20)

Characteristic	No. (%)
Sex, female	9 (45)
**Years post residency**	
0 (still in residency)	2 (10)
1–3 y	3 (15)
4–9 y	9 (45)
10–14 y	0 (0)
≥15 y	6 (30)
**Setting**	
Urban	9 (45)
Suburban	7 (35)
Rural	4 (20)
**Type of ED**	
Community	13 (65)
University	4 (20)
Government system	3 (15)
**Teaching vs nonteaching ED**	
Teaching	11 (55)
Non-Teaching	9 (45)
**Average annual ED volume**	
$20,000–39,999	4 (20)
$40,000–59,999	3 (15)
$60,000–79,999	6 (30)
$80,000–99,999	5 (25)
$≥100,000	2 (10)
**US geographic region**	
Midwest	4 (20)
Northeast	3 (15)
South	8 (40)
West	5 (25)

### Barriers

We identified barriers to optimal antibiotic prescribing within the person (provider and patient), task, organization, tool and technology and external-environment work-system elements of the SEIPS framework (Table 2).

**Table 2.** Barriers to Optimal Antibiotic Prescribing, Corresponding Work System Element, Infection Type and Representative Quote

Barriers	Primary Work System Element	Secondary Work System Element	Infection Type	Representative Quote
Patient expectations	Person	Task	Both^a^	Q1. Everybody’s expectation is that they are treated with some type of antibiotic. So as far as cellulitis goes, I do give them antibiotics… I don’t agree with it, and I try to educate them, I try to do that. But, unfortunately, you can spend 20 minutes educating someone and they’ll still say, “Well, where’s my prescription?” (EP017)
				Q2. There is some component of feeling pressured by the patients to do something about it and not just say, “Put a warm compress,” or “Take Tylenol or Motrin.” (EP001)
High-risk patient profile	Person	Task	Both	Q3. If it’s recurrent, if it looks bad, and you’re going to send them out, then we’ll put them on a [dual therapy]. (EP008)
				Q4. Usually, I will err on the side of caution to treat if they have other risk factors, especially for a diabetic. (EP006)
Provider fear of adverse outcomes	Person	External environment	Both	Q5. I would say that there are patients that I don’t think the antibiotics are really going to help, but I’ll still put them on because of risk of bounce back [relapse] or risk of it getting worse. (EP008)
				Q6. I think probably the biggest one is the fear of progression to sepsis … so the bounce back [relapse] of a patient who you discharged with a significant cellulitis and came back septic, and, you know, diabetic and dies from septic shock. (EP014)
Prioritization of proximal complaint over potential consequences of antibiotics	Person	External environment	Both	Q7. So the general public theoretical concern is of no interest to me… I only have one responsibility when I’m taking care of a patient, and that’s the patient. That’s it. (EP013)
				Q8. How do you weigh your decision on an individual patient versus 10 years from now? So the answer is, sure, it does bother me at times, absolutely but my immediate concern is the patient. I mean we don’t always do the right thing for the patient and we overprescribe, absolutely. (EP012)
Diagnostic uncertainty	Task	Person	Cellulitis	Q9. I think, the 2 big questions are, am I going to treat this, and then what coverage am I going to select? Physicians aren’t great at telling if it’s cellulitis or not, and often we end up 60% or 70% sure something is cellulitis or maybe even less so… Often we will treat it as cellulitis even if we’re not particularly convinced this is 100% cellulitis. So I think the treatment threshold is relatively low for providing antibiotics. (EP018)
		Organization		Q10. If I’ve ruled out every other alternate diagnosis, and cellulitis is what’s left, I would put them on antibiotics and have them follow-up with their primary doctor in a few days to see if it’s improved. (EP019)
		Person		Q11. Those cases where it looks like it, and I’m worried about it, and I’ll treat it, but and because we don’t have any definitive tests, and you have to use your clinical judgment. But that’s always in the back of my mind. Does this patient really need this? (EP005)
No clear diagnostic test	Task	Tools & technology	Cellulitis	Q12. Just keep in mind, it’s very subjective… There’s no good lab study out there to tell us one way or the other. (EP014)
Concern for MRSA	Task	External environment	Abscess	Q13. But unfortunately, the communities that I’ve always worked at, the MRSA is pretty high, so unfortunately, I usually go beyond just giving Keflex (EP001)
		Person	Abscess	Q14. You have to take into account the risk of the patient. If they’re immunosuppressed diabetic, on and on and on, then and the other factor is whether you suspect it to be a MRSA. If it’s a recurrent abscess, cutaneous abscess more suspicious of MRSA, then I might treat it. (EP013)
Unacceptable to be wrong	Organization	Task	Both	Q15. The concern that, you know, the patient may get worse. It’s all a guess. And so, you know, we’re not allowed to be wrong ever. It’s not acceptable ever to be wrong. (EP013)
Emphasis on patient satisfaction scores	Organization	Person	Both	Q16. I feel sometimes like I am overprescribing. And as I mentioned before, I feel sometimes a lot of pressure from the patients and administration to prescribe when in fact the patient doesn’t really need it. (EP001)
				Q17. If it looks like a little small nothing that you’re opening it up, I may not give them or you may give them Keflex because they’re not satisfied, you know. It’s like I don’t know if they use Pres Ganey where you are, but then they give you a bad Pres Ganey because you didn’t give them an antibiotic. (EP004)
Access to care	Organization	External environment	Both	Q18. … But with primary care the way it is and with patients without insurance, yeah, it’s concerning. So I’m going to err on the side of treatment. (EP012)
Time pressures/ ED crowding	Organization	Person	Both	Q19. If you don’t have time, because you’re in a very busy ER with, you know, hall beds and people in the waiting room. I feel like it becomes a secondary thing, and people have an expectation and taking the time to convince them that they don’t need it becomes challenging. (EP015)
Rapid diagnostics	Tool & technology	Internal Environment	Abscess	Q20. If I had like a rapid PCR for MRSA, I’d use that, or if we, even a nasal swab, you know, that came back. The problem is, in an emergency setting, it would have to come back in a rapid fashion. I wouldn’t use it on every patient, but on my patients where I had clinical uncertainty, I would definitely rely on that. (EP020)
More definitive diagnostic tests	Tool & technology	Task	Cellulitis	Q21. I think we would probably love if there was some sort of, as close to definitive as you can get, way of figuring out whether it truly a cellulitis or whether this is not a cellulitis. (EP014)
Standard of practice	External environment	Task	Both	Q22. I think there is the barrier of almost expectation, not only from the patient but from like a historical standpoint. I mean, you know, you have what appears to be or is concerning for cellulitis, that is something that people treat with antibiotics, and I think that’s just the known historical thought. I think there’s almost a point of like a standard of care. (EP015)
Equipoise in the literature	External environment	Task	Abscess	Q23. I think if someone requested or demands it, I’ll have a talk with them about the risks and benefits. You know, again because it’s such an area of equipoise with the abscesses, it’s hard for me to say like, if I’ve explained risks and benefits and somebody is like, I don’t care about diarrhea, I want to make 100% sure this doesn’t come back, I’m willing to take the risk and take antibiotics. (EP018)

a Both includes cellulitis and abscess.

#### Person-level barriers

Physicians described how patient expectation of treatment for both cellulitis and abscess was a barrier to optimal antibiotic prescribing because it led physicians to give patients antibiotics even if they did not always think they were necessary (Q1, Q2). Physicians described an increased willingness to prescribe antibiotics and to prescribe multiple antibiotics for SSTIs for patients who have an increased risk profile (eg, diabetes, recurrent infections), even if there was considerable diagnostic uncertainty (Q3, Q4). Providers also described how provider fear of treatment failure, including the development of a more serious infection with delayed treatment (eg, sepsis) and relapses were barriers to optimal antibiotic prescribing (Q5, Q6). Concerns over the chance of treatment failure were prioritized over the potential harms related to unnecessary antibiotics (Q7, Q8).

#### Task-level barriers

Diagnostic uncertainty was one of the primary barriers to optimally utilizing antibiotics for cellulitis. Physicians described utilizing antibiotics for a suspected cellulitis even if they had low levels of diagnostic certainty (Q9–Q11). Physicians described how optimal antibiotic usage for cellulitis was challenging because there is no objective diagnostic test (Q12). For abscess, diagnostic certainty was not a barrier except as it related to not knowing whether the causative organism was methicillin-resistant *Staphylococcus aureus* (MRSA). This uncertainty often led to the prescription of multiple antibiotics to achieve expanded spectrum of coverage (Q13, Q14).

#### Organization-level barriers

Physicians described an organizational culture where it is unacceptable to miss a bacterial infection (Q15). This culture encouraged physicians to ‘err on the side of caution’ and prescribe in cases of diagnostic uncertainty. Specifically, physicians cited pressure from hospital or department administration to ‘do something’ for patients as being a barrier to optimal antibiotic utilization (Q16). This pressure was particularly apparent when providers’ institutions emphasized patient satisfaction scores and if the provider believed the patient expected antibiotics (Q17). Physicians described poor access to ED follow-up care as a barrier to optimal antibiotic utilization because in many cases they could not count on a patient being seen for reevaluation in a day or 2 and were thus more likely to treat these patients with antibiotics in the ED (Q18). Finally, physicians described how time pressures in a busy ED was a barrier to optimal antibiotic prescribing because they simply did not have time to talk with patients about appropriate antibiotic use, including risks and benefits (Q19).

#### Tool and technology-level barriers

Physicians also described the need for diagnostic tools and how the absence of these tools made it hard to optimally diagnose SSTIs. For abscess, physicians were interested in having a rapid diagnostic test that could detect the presence of MRSA (Q20). Likewise, for cellulitis, physicians described the need for new tools to help them accurately diagnose cellulitis (Q21).

#### Environment-level barriers

We identified several external environment barriers to optimal antibiotic prescribing. Many physicians sensed that the current standard of practice in EM is to utilize antibiotics to treat cellulitis if there is any degree of clinical suspicion and indicated that it is challenging to go against historical standard of practice (Q22). Likewise, with abscess, many physicians described equipoise in the literature regarding the optimal utilization of antibiotics, which can make it hard for physicians to know how to optimally utilize antibiotics (Q23).

### Facilitators

The physicians also described person and task-level facilitators (Table 3).

**Table 3.** Facilitators to Optimal Antibiotic Prescribing, Corresponding Work System Element, Infection Type and Representative Quote

Facilitators	Primary Work System Element	Secondary Work System Element	Infection Type	Representative Quote
Shared decision-making conversation	Person	Organization	Both	Q24. So I would say that most of the time in my primary job, I can get buy-in for the mimic and I can also get buy-in for “we’re going to give this a trial” if I actually have time to talk to patients. So I try to talk about diarrhea, *C. diff*, and yeast infections, and that’s why we try to hold off. Plus, if you get this again and it is a cellulitis, then we really want to be able to have the antibiotic for you at that point in time. (EP015)
Identifying as a steward of antibiotics	Person	Task	Both	Q25. I think I’m a pretty good steward of antibiotics… giving the right antibiotic for the right thing and not giving antibiotics when they’re not indicated. (EP002)
				Q26. People get recurrent MRSA abscesses, and they get an antibiotic every time. But I’m actually more concerned about that patient, because they’re at risk for developing resistant organisms to the very drugs that they may need in the future, you know, when they become elderly and immunosuppressed and diabetic and things. So I will actually be closer stewards of antibiotics in their case, and if it’s a discrete abscess, be like I really don’t want to put you at risk for drug resistant organisms. (EP020)
Explaining the potential for I+D alone to cure	Task	Internal environment	Abscess	Q27. I have the time to do the I+D, which is going to fix them. So most of the time, you explain it to them. They’re just happy that the thing is gone. That’s their ultimate goal. (EP002)
Considering cellulitis mimics	Task	Person	Cellulitis	Q28. So you’ve got the textbook, right, redness, warmth, venous tracking, fluctuance or signs of abscess, fever, systemic illness. So that’s kind of the basis, the basic level. And then you’ve got the patient in front of you, who didn’t read the textbook and could have any mixture of those symptoms or partial symptoms. And my initial approach is to really make a commitment to whether I think this is cellulitis or not, and that’s based on ruling out mimics. And I just want to, I guess it’s a process of elimination saying, okay, I don’t think this is a mimic. I think it’s a cellulitis. (EP020)
Watchful waiting	Task	Organization	Both	Q29. Yeah sometimes, I think it is not as likely a cellulitis… If we’re doing kind of like a watchful waiting with someone that has a very early case of skin irritation, then if I know they have a primary care doctor, they can go there. If they don’t, I just have them come back to the ER. (EP007)
Wait-and-fill prescription	Task	Person	Both	Q30. If someone comes in and feels very, very strongly that they need antibiotics and I don’t feel like they do, I’d probably use the strategy of here’s a prescription. Please wait a day, see if it progresses, and then you can use it. (EP019)

#### Person-level facilitators

Many physicians described having a shared decision-making conversation with patients. They felt that if they had enough time, they could often obtain buy-in to plans that did not involve prescribing an antibiotic (Q24). This finding contrasts with the patient-expectation barrier described previously, in which many physicians felt like patients always expected antibiotics no matter how much they discussed the idea of not prescribing with patients. A second person-level facilitator was physicians who self-identified as ‘antibiotic stewards.’ These physicians expressed the importance of antibiotic stewardship and in cases of uncertainty were more likely to consider the risk to benefit ratio related to antibiotics (Q25, Q26).

#### Task-level facilitators

Physicians described many task-level facilitators that helped them optimally prescribe antibiotics for skin infections. For abscess, physicians described how they could routinely convince patients that they did not need an antibiotic after completing an incision and drainage because they had done an intervention, drained the infection (Q27). For cellulitis, ruling out mimics was a facilitator that physicians used to help them optimally use antibiotics (Q28). Additionally, physicians described using the facilitator, watchful waiting, where they would not give a patient an antibiotic but instead put in place a plan for a recheck if the infection worsened (Q29). Finally, some physicians described providing a wait-and-fill prescription in which they would prescribe an antibiotic only to take under certain circumstances (eg, expanding erythema) (Q30).

### Intervention mapping

We selected modifiable barriers and operationalizable facilitators and strategies identified by the physicians, and developed proposed interventions that could mitigate the barrier or enhance the facilitator (Table 4). Each intervention has a detailed description that underwent multiple rounds of refinement using input from a multidisciplinary group of stakeholders. These interventions cut across many of the identified SEIPS work-system elements, and they address several concerns: lack of access to ED follow-up care, patient expectations, diagnostic uncertainty (eg, MRSA and pseudocellulitis); fear of adverse outcomes, perceived clinical equipoise, and provider knowledge gaps. They range from systems-level programs (eg, community paramedicine follow-up programs) to novel diagnostics and clinical decision support tools.

**Table 4.** Mapped Skin and Soft-Tissue Infection Stewardship Interventions for the Emergency Department

Barrier	Infection Type	SEIPS Work System Element(s)	Mapped Intervention	Intervention Description
Lack of access to ED follow-up care	Both	Organization and external environment	Telehealth or community paramedicine program for reliable outpatient follow-up	The emergency department can arrange 24-hour follow-up for discharged patients by either an in-home visit by a community paramedic or a virtual appointment using an online, video enabled telehealth system.
Patient expectations	Both	Person	Exclude encounters involving inappropriate antibiotic requests from satisfaction metrics	Your hospital quality department allows you to flag cases involving inappropriate requests for antibiotics, and these are excluded from your patient satisfaction metrics.
Diagnostic uncertainty (MRSA)	Abscess	Tools and technology, tasks	MRSA PCR of purulent infections	Your laboratory offers rapid (∼90 minute) turnaround time for assay capable of detecting MRSA in purulent material from the I+D procedure negative predictive value of 95%.^32^
Diagnostic uncertainty (pseudocellulitis)	Cellulitis	Tools and technology, tasks	Clinical decision score (ALT-70) and/or thermal imaging camera	A thermal imaging camera indicates the maximum skin surface temperature of the affected leg is identical to the unaffected leg. The average reported skin temperature difference for cellulitis is 3.7°C greater in the affected limb.^33^
Fear of adverse outcomes	Both	Person, external environment, organization, internal environment	Shared decision making tool	The tool will facilitate a more efficient, less time-consuming conversation about risks and benefits of antibiotics for the particular clinical scenario.
Perceived clinical equipoise	Abscess	Person, tools and technology, tasks, organization	Clinical decision support/Best-practice alert	Your electronic health record has alerted you that this condition can potentially be managed without antibiotics in the majority of cases [cf, number needed to treat (NNT) with antibiotics to prevent 1 treatment failure = 14–26]. No serious complications observed in placebo group of uncomplicated abscess trials.^28–30^
Provider knowledge gaps	Cellulitis	Person, tools and technology, tasks, organization	Clinical decision support/Best-practice alert	A best-practice alert in the electronic health record has triggered the following message, “Studies indicate up to 30% of cellulitis cases diagnosed in the emergency department are actually misdiagnosed mimics which do not require antibiotics.”^2,3^

## Discussion

In this analysis, we present the first qualitative assessment of perceived barriers and facilitators to optimal antibiotic prescribing for SSTIs from the perspective of emergency physicians. Utilizing the SEIPS systems engineering framework enabled us to identify barriers beyond the patient and provider themselves. This process directly addresses calls to develop quality improvement interventions (eg, antibiotic stewardship) that are grounded in systems engineering and behavior change theory and that are informed by data collected from frontline providers.^9,25–27^ Key identified barriers to optimal antibiotic prescribing for SSTIs included poor access to follow-up care (organization), need for more definitive diagnostic tools (tools and technology), and fear over adverse outcomes related to missed infections (person).

One unexpected finding of our analysis was the identification of knowledge gaps and skepticism of the literature. For instance, many providers held the view that antibiotics should now be given to all patients with uncomplicated abscesses based on recent trial data.^28,29^ There was a lack of awareness about the high number needed to treat in these trials and recent calls for a more nuanced approach to antibiotic prescribing for uncomplicated abscesses.^30,31^ Additionally, most providers doubted the validity of literature citing a 30% misdiagnosis rate of cellulitis in the ED.^7^ One potential technological solution to these knowledge gaps would be clinical decision support embedded in the electronic health record as best-practice alerts.

SSTIs pose a particular diagnostic challenge given the absence of a gold-standard test. Providers expressed that the treatment decision must be made despite significant diagnostic uncertainty. Most providers opted to ‘err on the side of caution,’ which involved prescribing an antibiotic(s) even if the perceived likelihood of bacterial infection and/or their diagnostic certainty was low. This was especially true when other barriers were present such as poor access to follow-up care or a high-risk patient profile, which essentially lowered the bar to prescribe an antibiotic. The perceived patient safety and professional risk related to failing to provide antibiotics for an actual SSTI typically outweighed the acknowledged risk of adverse drug reactions and detrimental impact on public health related to unnecessary antibiotic use. Providers felt that evidence-based diagnostic tools that would make the SSTI evaluation process more objective would enable them to avoid prescribing in cases of low clinical suspicion.

Interestingly, several evidence-based interventions would fit this need that have not been extensively studied or adopted. For instance, rapid MRSA assays for purulent infections that strongly correlate with traditional cultures and improve tailored prescribing have been available for years.^32^ Although not as well established, risk stratification scores (ALT-70) and surface thermal imaging have demonstrated potential to accurately differentiate cellulitis from pseudocellulitis.^33,34^

The perception among providers that patients generally expect antibiotics has been documented across a variety of healthcare settings, including the ED.^11,35^ However, research examining the expectations of patients with respiratory tract infections (RTIs) in the ED did not find that patients routinely expect antibiotics.^11,36^ Although RTIs are a distinct clinical syndrome when it comes to antibiotic decision making, our findings suggest that a perceived expectation of antibiotics also plays a role in SSTIs. In addition to encouraging providers not to assume patients expect an antibiotic, a potential intervention would be for healthcare organizations to exclude encounters involving demands for nonindicated antibiotics from patient-satisfaction metrics. Alternatively, a more patient-centered approach towards education and shared decision making could potentially avoid this issue altogether. The development of a shared decision-making tool to facilitate patient–provider communication, such as has been demonstrated effective in reducing low value workups for low-risk chest pain in the ED, could enable clarification of the patient’s actual expectations (if any) while educating them about their individual risk and the providers level of diagnostic certainty (or lack thereof).^37,38^

Barriers related to the external environment need to be addressed at a healthcare-system level. For instance, providers often ‘lower the bar’ to treat patients who have known difficulties with access to follow-up care. Ensuring that the patient could have a repeat assessment in a timely fashion to ensure any progression of the condition is identified as soon as possible could increase provider comfort in withholding antibiotics. With the rapid expansion of telehealth services due to the COVID-19 pandemic, it is more feasible than ever to incorporate either synchronous or asynchronous follow-up visits into ED SSTI care protocols.

This study had several limitations. Our recruitment strategy was an opt-in system, and it is possible that physicians who were already informed and interested in managing infections in the ED were the participants in the study. Because the primary aim of this analysis was to identify unifying themes, it is important to note that our findings do not represent an exhaustive set of emergency physician perspectives on this phenomenon. The proposed interventions were based on a mapping process guided by the identified themes, but we did not attempt to ascertain the magnitude of their potential impact or feasibility in different practice settings.

Using a systems engineering informed qualitative approach, we were able to characterize a number of barriers and facilitators to optimal antibiotic use for SSTIs specific to the ED work system. The developed mapped interventions span multiple components of the ED work system and should inform future efforts to improve antibiotic stewardship for SSTIs in this setting.

## Supplementary material

For supplementary material accompanying this paper visit https://doi.org/10.1017/ash.2022.316 click here to view supplementary material
