# Supplementary material for: Characterizing barriers to antibiotic stewardship for skin and soft-tissue infections in the emergency department using a systems engineering framework
Source: Antimicrob Steward Healthc Epidemiol. 2022 Nov 7;2(1):e180. doi: 10.1017/ash.2022.316 (PMC9641503; doi:10.1017/ash.2022.316)
Supplement: Supplementary file 1 [file ashsup.zip › S2732494X22003163sup001.docx]

**Qualitative Interview Guide**

**Diagnosis:**

1. Describe your general approach to diagnosing suspected **cellulitis** in the ED. Walk me through your process.

2. What diagnostic tests, if any, do you use in guiding the diagnosis of a suspected **cellulitis** in the ED?

3. What factors do you consider when you are deciding to treat a suspected **cellulitis**?

4. How do you decide whether to give someone antibiotics for suspected **cellulitis**?

5. Describe your general approach to diagnosing skin and soft tissue **abscesses** in the ED.

6. What diagnostic tests, if any, do you use in guiding the diagnosis of **abscesses** in the ED?

7. What other types of resources or references, if any, do you use to assist in the diagnosis of skin and soft tissue infections **(cellulitis or abscess)**?

8. What factors do you consider when you are deciding to treat an **abscess**?

9. How do you decide whether to give someone antibiotics for an **abscess**?

*Now I am going to ask you questions about how you decide on a disposition for patients with skin and soft tissue infections in the ED*

**Disposition:**

10. How do you make disposition decisions for patients with skin and soft tissue infections in the ED (admit vs discharge)? What specific factors influence this decision-making process? Are there specific barriers to management of **suspected** skin and soft tissue infections without antibiotics (discharge or admission observation trial)?

*For this last section I am going to ask you to think about your decision to use antibiotics throughout the entire process of treating patients with SSTIs, including diagnosis, treatment, and disposition.*

**Specific Factors:**

11. Are there any additional resources that could be provided to assist you in the optimal use of antibiotics in the management of skin and soft tissue infections in the ED?

12. Is there ever a scenario when patient load or ED census (crowding) could influence your antibiotic decision making for skin and soft tissue infections?

13. Does the ED staffing model ever influence antibiotic prescribing?

14. If you work with APPs, how often do you personally evaluate their patients? Does that vary for cases involving antibiotic prescribing? How do you approach it when someone under your supervision wants to prescribe an antibiotic?

15. Do you have a formal patient follow up process in your ED? How does this impact patients treated with antibiotics for skin and soft tissue infections? How does this impact patients discharged without antibiotics for potential infections?

16. Please describe your EHR system. Does your EHR include any decision support tools involving infectious diseases? If so, describe what they are, how they work, and how you typically interact with them. Do you find them useful?

17. Does your ED have a process by which you can coordinate care with outpatient providers? Do you utilize this for patients being treated for confirmed or potential skin and soft tissue infections?

18. Does your primary hospital have an antibiotic stewardship program? Please describe it. Does it have an ED focused component? Please describe that. Has it done any interventions targeting skin and soft tissue infections in the ED? If so, please describe. Does your organization monitor antibiotic prescribing (auditing)? If so, please describe how this information is relayed to you (benchmarking, quality review, etc.).

19. Do patient level factors influence your antibiotic prescribing for skin and soft tissue infections? How do these patient level factors that you have identified influence your disposition decisions for patients with skin and soft tissue infections?

20. Do you use any rapid diagnostic assays or biomarkers to aid in the diagnosis of skin and soft tissue infections in the ED? If so, which ones do you use and how do you use them?

21. Are there any decision support tools that you use? Do you find them helpful? What would make them more helpful?

22. Does your emergency department have its own antibiogram? If so, do you know how to access and use this resource? If so, how do you use this resource (scenario, how often)?

23. How do available best practice guidelines regarding the management of skin and soft tissue infections influence your practice, if at all?

24. How do you go about keeping your knowledge on appropriate treatment for skin and soft tissue infections up to date?

25. Now I am going to read you a statement and I am curious what you will think about it: Studies estimate somewhere between 30-50% of cellulitis diagnoses in the ED incorrect, what do you think about this statement?

26. Do you feel there are downsides to antibiotic prescribing? If so, what are they? How do you balance these downsides in clinical practice?

27. What are the principal barriers to optimal antibiotic use in the management of skin and soft tissue infections in the ED?

28. Is there anything else you would like to add related to skin and soft tissue management in the ED?
