# Supplementary material for: Characterizing barriers to antibiotic stewardship for skin and soft-tissue infections in the emergency department using a systems engineering framework
Source: Antimicrob Steward Healthc Epidemiol. 2022 Nov 7;2(1):e180. doi: 10.1017/ash.2022.316 (PMC9641503; doi:10.1017/ash.2022.316)
Supplement: Supplementary file 1 [file ashsup.zip › S2732494X22003163sup002.docx]

| **Coding definition** |
| --- |
| 1. **Barrier/Facilitator Codes** |
| **A1. Barrier:** A characteristic of a work system element(s) that interferes with a person’s ability to accomplish a goal or an activity efficiently, appropriately, and accurately. |
| **A2. Facilitator:** A characteristic of a work system element(s) that makes it easy or easier to accomplish a goal or activity efficiently, appropriately, and accurately. |
| **A3. Description:** Segment of text that does not explicitly talk about a facilitator or a barrier but is describing the process in a neutral manner. |
| 1. **SEIPS Codes** |
| **B1. External Environment:** Regulatory, legislation, medical/legal environment, or societal factors that are external to healthcare system. |
| **B2. Internal Environment:** The physical environment in which the diagnosis and treatment decisions are made. This includes the emergency department layout, temperature, noise levels, crowding, observation unit, boarding patients |
| **B3. Organization:** The larger context in which the diagnosis and treatment decisions are made. Including, the hospital or primary care culture, policies and procedures, work schedules, and relevant training provided. Local healthcare environment, treatment algorithms. |
| **B4. Person:** Characteristics including the education, skills, motivation and knowledge of the healthcare team, individual healthcare professional, patient and/or caregiver. |
| **B5. Tasks:** The individual activities performed during the diagnosis and treatment of infection (e.g., evaluating the patient, deciding on a treatment, deciding on a disposition, administering the treatment, documenting the process in the EHR). |
| **B6. Tools and Technology:** Any tools or technology the healthcare worker uses in the diagnostic and treatment process. |
| 1. **Diagnosis and Treatment Process Codes** |
| **C1. History of Present Illness:** Information about circumstances that led patient to seek emergency care. This includes timing, duration, and symptom descriptions. |
| **C2. Past Medical History**: This includes any preexisting conditions (comorbidities) and history of similar conditions which are relevant to current presentation. |
| **C3. Family history**: Any aspects of the patient’s family medical history or medical conditions involving close contacts which are relevant to the current presentation. |
| **C4. Social history**: Any living situation or substance use issues relevant to the current presentation. |
| **C5. Physical examination:** Any descriptions of elements of the physician’s evaluation that include auscultation (listening), palpation (touch), visualization, or smell. Also, any references to vital signs, infection size and location. |
| **C6. Laboratory testing**: Diagnostic testing involving blood samples. |
| **C7. Cultures**: Previous culture results or collection of new clinical specimens for the purpose of infectious organism identification. |
| **C8. Imaging**: Diagnostic testing involving the use of x-rays, CT scans, ultrasound, or MRI. |
| **C9. Molecular diagnostics**: Non-culture diagnostic testing involving clinical samples for the purpose of infectious organism identification. |
| **C10. Diagnosis (Final):** The final diagnosis that the clinician makes or the process that leads to the final diagnosis |
| **C11. Drainage procedures**: Any intervention aimed at draining a skin infection to include needle aspiration, manual expression, or incision and drainage |
| **C12. Post drainage procedure management:** Any part of the procedure to drain a skin infection which occurs after the needle insertion or incision portion is complete. E.g., irrigation, packing, blunt dissection, loop placement |
| **C13. Decision to treat skin infection with antibiotic:** Discussing the prescribing decision for treating skin infections with antibiotics |
| **C14. Selection of antibiotic (s):** the actual selection of a specific antibiotic(s) for this infection, may include consideration of potential causative organism, patient allergies, past medical history, etc. |
| **C15. Selection of antibiotic dose:** the actual selection of a specific antibiotic(s) dose for this infection, may include consideration of potential causative organism, patient allergies, past medical history, etc. |
| **C16. Selection of antibiotic duration:** duration for this infection, may include consideration of potential causative organism, patient allergies, past medical history, etc. |
| **C17. Disposition decision**: how physician decides whether to discharge patient home versus placing in observation (ED) or inpatient admission. |
| **C18. Patient Adherence:** Discussions around filling prescriptions and taking medication as prescribed |
| **C19. Post discharge instructions and follow up**: The information given to patients upon discharge tells them how to manage their condition and follow-up for subsequent care. Includes providing reasons to return to the ER (signs of worsening infection). |
| 1. **Infection Type Codes** |
| **D1. General**: Talking about skin and soft tissue infections generally (not specific to any of below) |
| **D2. Cellulitis**: Specifically talking about cellulitis |
| **D3. Abscess:** Specifically talking about abscess |
| **D4. Purulent Cellulitis:** Abscess with overlying/surrounding cellulitis |
| **D5. Necrotizing Fasciitis:** Specifically talking about necrotizing fasciitis. |
| **D6. Osteomyelitis:** Specifically talking about osteomyelitis |
| 1. **Summary Codes** |
| **E1. ANTIBIOTICS/TREATMENT:** Group of codes that describe how providers use antibiotics and how they conceptualize treatment. |
| **E1a. Antibiotic Characteristics:** Physicians describe specific characteristics of antibiotics such as drug to drug interactions, safety profile, adverse side effects, and cost. |
| **E1b. Antibiotic Shortages:** Issues with supply chain of antibiotics which make them unavailable and change prescribing behavior. |
| **E1c. Antibiotic Stewardship:** Physician mentions anything about antibiotic stewardship |
| **E1d. Dose before disposition** Providers describe giving an IV dose of antibiotics before discharge or admission to facilitate treatment or for other reasons. |
| **E1e. Just in Case/Err on side of caution:** Antibiotics that are used just in case there is an infection. |
| **E1f. Previous Treatment:** physician describes how a previous successful treatment successful or treatment failure in a patient influences diagnostic and treatment decisions such as antibiotic prescription, inpatient vs. outpatient treatment or diagnostic testing. |
| **E1g. Spectrum Antibiotics:** Physicians mention wanting to use antibiotics with a specific spectrum of activity. |
| **E1h. Standard of Practice:** If I am doing what the rest of my group is doing, I will be okay, cannot go too outside the lines |
| **E1i. Topical antibiotics/decolonization:** Providers discuss applying antibiotics to either nose or skin to eliminate MRSA. Also includes dilute bleach baths. |
| **E1j. Wait and Fill Prescription:** Physicians thoughts regarding the idea of giving a prescription and then having the patient wait to fill it depending on clinical parameters of disease progression. |
| **E2. Diagnosis:** Group of codes that describe the diagnostic process. |
| **E2a. Clinical Diagnosis:** Physicians state that the diagnosis of skin and soft tissue infections is clinical. |
| **E2b. Diagnostic Uncertainty/Mimics:** Concept of not being sure of a diagnosis is discussed. Physicians describe alternate diagnosis that may mimic cellulitis or abscess |
| **E2c. MRSA:** Physicians mention MRSA usually as it pertains to prevalence or how it influences patient care. |
| **E2d. Sepsis/Systemic Illness/SIRS:** References to manifestations beyond skin and soft tissue infections or being acutely ill/unstable. |
| **E2e.Size or Location of Infection:** Provider mentions how size or location of infection influences diagnosis, treatment, or disposition. |
| **E3. PATIENTS:** Characteristics inherent to the patients themselves. |
| **E3a. Age:** Provider references how patient age influences care. |
| **E3b. Allergies:** Provider references how patient allergies influence care. |
| **E3c. Comorbidities:** Physicians mention any type of comorbidity as influencing care. |
| **E3d. Funding/Insurance/Financial Issues:** Providers discuss how a patient’s insurance or financial limitations influence care. |
| **E3e. Pain:** Providers talk about how a patient’s degree of physical pain influences care. |
| **E3f. Patient and Family Expectations:** Patients or family members that have expectations of a certain quality of care (e.g., antibiotic prescribing or being discharged) when they go to the ED |
| **E3g. Dependable Patient:** Physicians describe what does and does not make a dependable patient (e.g., concern for non-compliance, issues with health literacy). |
| **E3h. Social Factors:** includes substance use, homelessness, etc. |
| **E3i. Shopping Around for Antibiotics:** Physicians talk about how if they do not give an antibiotic, the patient will just go elsewhere to get one. |
| **E4. PROVIDERS:** Group of codes that describe provider behavior and how it influences care. |
| **E4a. Communication Strategies:** Providers discuss how communication issues influence patient care. |
| **E4b. Litigation:** Physicians mention concern or lack of concern regarding malpractice litigation. |
| **E4c. Negative Outcome:** Tendency for physicians to remember negative events such as treatment failure because patients come back into ED, they get a reprimand, etc. and discussion of fear of negative clinical outcome |
| **E4d. Physician Characteristics:** Self-described **c**haracteristics that the physicians have that make diagnosis and treatment of skin and soft tissue infections easier or harder. |
| **E4e. Residency/Training:** Providers reference what they were taught or how they behaved when they were a resident |
| **E5. OTHER PROVIDERS:** Group of codes that are used when providers mention how other health care professionals influence patient care in the ED. |
| **E5a.** **Hospitalists:** may be referred to as admitting doctors/team or internal medicine/family medicine doctors/team |
| **E5b.** **Infectious Disease**: ID specialty. |
| **E5c. Nurses/APPs/Social workers/Case management**: Providers mention role of nurses, APPs or social workers |
| **E5d. Pharmacists**: Providers mention the role of pharmacists either ones in the ED or elsewhere |
| **E5e.Surgeons:** Providers mention the role of surgeons. |
| **E6. System/Tools:** Group of codes that describe system level factors that influence patient care. |
| **E6a. Accessibility:** Ability for patients to get the timely care they need after discharge from the ED. |
| **E6b. Administration:** Providers describe how pressure from administrators or hospital policy influences behavior. |
| **E6c. Antibiogram**: Mentions the Antibiogram of an ED or the institution. |
| **E6d. ED Care Setting:** When provider discusses some unique aspect of the ED practice setting in general terms. |
| **E6e. ED Crowding/Time Pressures:** Providers mention how ED crowding (boarding), or time pressures influence patient care. |
| **E6f. Electronic health record (EHR):** including issues of order entry, documentation, decision support, or diagnosis entry. |
| **E6g. Primary Care Doctor:** Physicians describe characteristics and role of primary care doctors in post-ED care. |
| **E6h. Guidelines:** Physicians talk about guidelines they use or the usefulness or non-usefulness of guidelines. Includes when provider specifically references a score that they utilize or know about for diagnosis/treatment of skin and soft tissue infections. |
| **E6i. Lower acuity care:** includes discussions about urgent care, fast track, and physician triage. |
| **E6j. Medical Records:** Mentions obtaining medical records for use in guiding care. |
| **E6k. Observation Status/Observation Unit:** Providers describe the role of observation status or an ED observation unit in patient care. |
| **E6l. References-specific:** Physicians give specific examples of references they use. |
| **E7. External Factors:** Societal or regulatory factors that are external to healthcare system. |
| **E7. Natural:** Providers describe how weather or seasonal variability influence pattern of disease (e.g., more cases of cellulitis in the summer). |
| **E7. Setting:** Provider describes how the setting where the ED is located or where the patients’ live influences care. Ex. Rural vs. urban |
| 1. **Miscellaneous** |
| **F1. Strategy:** Any method or process a healthcare professional or team uses to overcome challenges/barriers associated with diagnosing and treating infections. |
| **F2. Exemplar Quotes:** Quotes made by the participants that are descriptive or worth noting that may potentially be used to convey important aspects of the diagnosis and treatment of infection process. |
